# Shear and Flexural Behavior of RC Columns with Rectangular Hollow Cross-Sections Under Cyclic Loading

**DOI:** 10.3390/ma19061098

**Published:** 2026-03-12

**Authors:** Kiwoong Jin, Ho Choi, Chunri Quan

**Affiliations:** 1 Department of Architectural Engineering, Hannam University, Daejeon 34430, Republic of Korea; jink@hnu.kr; 2 Department of Architecture, Faculty of Science and Technology, Shizuoka Institute of Science and Technology, Fukuroi 437-0032, Japan; 3 Department of Architecture, Faculty of Engineering, Osaka Institute of Technology, Osaka 535-8585, Japan; chunri.quan@oit.ac.jp; 4 Graduate School, Osaka Institute of Technology, Osaka 535-8585, Japan

**Keywords:** reinforced concrete (RC) columns, rectangular hollow cross-section, seismic performance, flexural behavior, shear behavior, shear span ratio, cross-sectional analysis, FEM analysis

## Abstract

This study investigates the seismic behavior of reinforced concrete (RC) columns with hollow rectangular cross-sections through experimental testing and analytical evaluation. A series of hollow-sectioned column specimens with different shear span ratios and failure modes were tested under cyclic lateral loading to examine their flexural and shear performance. The results demonstrated that flexure-dominated columns exhibited stable load-deformation responses and sustained seismic performance, even with the reduced shear span ratio. The experimental results also showed that the presence of the hollow cross-section had minimal influence on flexural strength and bar strain distribution. The shear strength of RC columns with hollow cross-sections tended to be underestimated by the conventional equation; however, improved accuracy was achieved by applying an equivalent cross-sectional area in the calculation. A nonlinear finite element analysis successfully reproduced the flexural load-deformation response of the specimens. While some discrepancies were observed in the shear-dominated cases, the analytical model provided conservative estimates under positive loading. These findings provide new insights into the seismic design of RC columns with hollow cross-sections, highlighting their potential applicability in building structures when flexural and shear behaviors are properly considered.

## 1. Introduction

Reinforced concrete (RC) buildings generally exhibit high self-weight due to their material properties and the large dimensions of structural members. As a result, the seismic forces acting on such buildings can exceed those experienced by other structural systems. Given that seismic force is directly related to self-weight, reducing the self-weight is an effective strategy for minimizing seismic demand, particularly in RC structures. Therefore, employing hollow cross-sections is a promising solution for this purpose, offering not only structural efficiency but also potential economic and environmental benefits.

Hollow RC piers have been widely applied in bridge engineering, as they provide superior flexural stiffness and strength compared to their solid counterparts, without increasing mass. Accordingly, numerous studies have been conducted on the seismic performance of hollow circular bridge piers. For example, Chung et al. [1] investigated the effects of confinement ratios, axial loads, and retrofitting using glass fiber through quasi-static tests. Lee et al. [2] explored the relationship between axial force and deformation capacity under cyclic loading. Other researchers [3,4,5] examined various aspects such as load-bearing capacity and ductility. However, these studies predominantly focused on circular cross-sections intended for bridges, rather than building columns, and many used relatively low axial force ratios. Studies by Won et al. [6] and Liang et al. [7] also addressed circular hollow columns, but their loading conditions were limited to uniaxial compression.

In contrast, research on RC columns with rectangular hollow sections is still limited. Some work has been done; for instance, Calvi et al. [8] and Han et al. [9] evaluated structural behaviors under different axial loads and cyclic bilateral loading, respectively. Other studies [10,11,12,13,14] examined seismic capacity, damping characteristics, and analytical modeling of hollow rectangular sections in bridge and infrastructure elements. Nevertheless, reinforcement detailing and design considerations differ significantly between infrastructure and building components, limiting direct applicability. In addition, in conventional RC building design, self-weight reduction has typically been achieved by increasing material strength or reducing member dimensions [15]. For non-structural elements, techniques such as lightweight concrete and voided slab systems have been employed [16,17]. However, only limited research has focused on applying self-weight reduction techniques to primary structural members like columns and beams in buildings. Similar hollow or partially hollow column systems have also been applied in the reconstruction of important historical structures, such as the Basilica di Collemaggio in Italy, thereby demonstrating their practical feasibility in seismic regions [18]. It is also noted that, in most of the previous studies, although flexural strength and behaviors of hollow-sectioned columns have been discussed, there is almost no research on shear strength and behaviors by shear failure.

Therefore, in the previous study by the authors [19], the seismic performance of RC columns with rectangular hollow sections was investigated in detail through cyclic loading tests under compression axial force ratios ranging from 0.16 to 0.3. Additionally, a finite element (FEM) analysis was conducted to further understand their seismic behavior. In that study [19], the hysteresis responses and failure mechanisms of both solid and hollow-sectioned columns were compared, and the validity of using hollow-sectioned columns was confirmed. However, the test specimens had shear span ratios ranging from 2.79 to 3.17, which are considered relatively high for building structures. Herein, the shear span ratio is defined as 0.5*h*_0_/*d*, where *h*_0_ is the clear height of the column and *d* is the effective depth. Moreover, the focus of the previous study [19] was mainly on ductile behavior, in which the flexural failure is the dominant failure mode. Therefore, in this study, the seismic behavior of RC columns with rectangular hollow sections is investigated experimentally and analytically, under a reduced shear span ratio of 1.97 for wider application of the target structure. The shear strength and behavior of hollow-sectioned columns, where shear failure becomes the dominant failure mode, are also discussed in detail. It should be noted that, in this study, the shear span ratio was defined as 0.5*h*_0_/*d* to reflect the antisymmetric bending condition induced by mid-height lateral loading. Accordingly, this definition corresponds to half of the conventional *h*_0_/*d* ratio commonly adopted for cantilever-type column design.

## 2. Experiment Outline

This study is a follow-up to the authors’ previous work [19], in which the seismic behavior of RC columns with rectangular hollow cross-sections was investigated under relatively large shear span ratios. While a similar experimental framework was adopted, the present study focuses on columns with reduced shear span ratios to examine shear-dominated behavior and to clarify the applicability of hollow sections under different failure modes.

### 2.1. Specimen Design and Test Parameters

**Figure 1:**
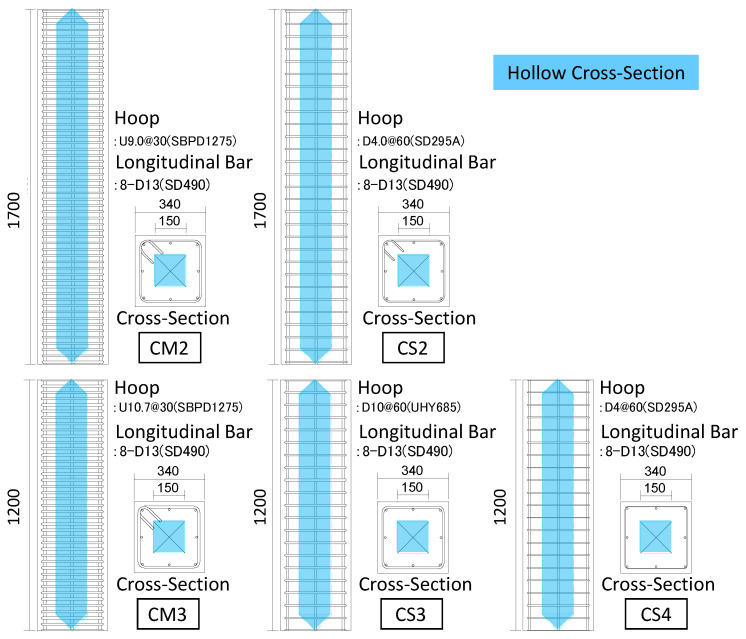
Details of the test specimens (Unit: mm).

Figure 1, Table 1 and Table 2 present the specimen properties, structural details, and material characteristics, respectively. Specimen CM2 was used in the previous study by the author [19], while specimens CS2, CM3, CS3, and CS4 were newly fabricated in this study. All specimens were 1/3-scale models, designed to represent middle-story columns in a 20-story high-rise residential building, assuming an axial force ratio (*η*) of 0.15. The axial force ratio (*η*) is defined as the ratio of the applied axial load to the nominal axial compressive capacity of the net concrete section, expressed as *η* = *N*/(*A_c_*·*F_c_*), where *A_c_* and *F_c_* represent the concrete cross-sectional area excluding the hollow core and concrete compressive strength, respectively. In this study, an axial force ratio of 0.15 was selected to represent typical middle-story columns in high-rise residential buildings, where gravity load effects are moderate compared to those at the ground level. Although ground-level columns may experience higher axial force ratios and consequently increased shear demand, the present study focuses on evaluating the applicability and prediction of shear capacity of hollow sections under representative story-level axial conditions.

Each column had a cross-sectional dimension of 340 × 340 mm, with a hollow core measuring 150 × 150 mm. As shown in Figure 1, the hollow sections consisted of square and tapered segments to help reduce stress concentration. The hollow section ratio, defined as the ratio of the hollow area to the total cross-sectional area, was set at 0.81 for all specimens. In addition, high-strength concrete (approximately 100 MPa) was adopted in this study to compensate for the potential reduction in flexural stiffness caused by the hollow section. The presence of the void inevitably reduces the second moment of area of the cross-section, which may lead to a decrease in initial stiffness. By using high-strength concrete with a higher elastic modulus, the stiffness reduction associated with the hollow core can be mitigated, thereby enabling a more realistic evaluation of structural performance.

**Figure 2:**
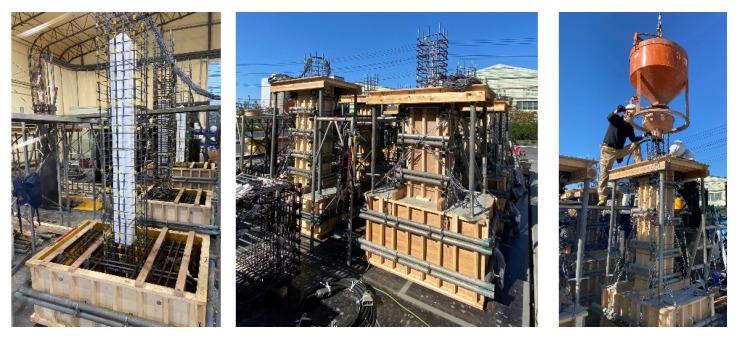
Specimen fabrication process.

The fabrication process of the specimens is illustrated in Figure 2. First, the longitudinal and transverse reinforcement cages were assembled in accordance with the design drawings. Strain gauges were attached to selected longitudinal and transverse reinforcing bars prior to casting to measure strain responses during cyclic loading. To reproduce the hollow core of the rectangular section, expanded polystyrene (EPS) foam blocks were inserted at the center of the column before concrete placement. The EPS core was securely fixed to prevent displacement during casting. Given that the mechanical stiffness of EPS is significantly lower than that of concrete, its structural contribution to stiffness and strength is considered negligible. The EPS element therefore functioned solely as a void former to reproduce the intended hollow cross-sectional geometry. After installation of the reinforcement and EPS core, wooden formwork was assembled, and concrete was cast in a vertical position. Mechanical vibration was applied during placement to ensure adequate consolidation. The specimens were demolded after initial curing and subsequently stored under standard curing conditions until testing.

As explained earlier, in the specimen CM2 used in the previous experiment, the shear span ratio was set at 2.79, and the specimen was designed to exhibit a flexural failure mode. In this study, specimen CS2 also had a shear span ratio of 2.79 but was designed to fail in shear by reducing the yield strength and spacing of the transverse reinforcement. In contrast, for the other specimens, CM3, CS3, and CS4, the column height was reduced, resulting in a shear span ratio of 1.97. The primary failure mode of CM3 was intended to be flexural, while both CS3 and CS4 were designed to fail in shear by reducing the yield strength and the shear reinforcement ratio.

Table 1 presents the calculated values of the ultimate flexural strength (*Q_mu_*) and the mean shear strength (*Q_su_*_,*mean*_) for all specimens. The flexural strength *Q_mu_* was evaluated through cross-sectional analysis under the plane section assumption by calculating the ultimate moment capacities at both the column ends and the fully hollow regions. In this process, the flexural strength was evaluated through sectional analysis based on the plane section assumption. The ultimate compressive strain of concrete at the extreme fiber was taken as 0.003, and an equivalent rectangular stress block was adopted following conventional reinforced concrete sectional analysis and widely used design provisions [19,20,21]. These assumptions are commonly employed in the evaluation of axial force–moment interaction behavior of reinforced concrete and composite columns. The constitutive behavior of concrete was modeled using the stress–strain relationship proposed by Popovics [22], whereas the reinforcing steel was represented by a bilinear stress–strain model.

The smaller of the two values, 2*M_u_*/*h*_0_ or 2*M_u_*′/*h*_0_′, was taken as *Q_mu_*, where *h*_0_ and *h*_0_′ represent the clear height of the column and the height between the fully hollow sections, respectively. For all specimens, *Q_mu_* was governed by 2*M_u_*/*h*_0_. *Q_su_*_,*mean*_ was calculated using Equation (1), proposed by Arakawa [20], which is commonly employed for estimating shear capacity in Japan. To account for the effect of the hollow section, the effective column width (*b*′) excluding the hollow portion was used in the calculation of *Q_su_*_,*mean*_. As shown in Table 1, the ratio *Q_su_*/*Q_mu_* (shear strength margin) exceeds 1.0 for specimens expected to exhibit flexural failure, whereas it falls below 1.0 for those designed to fail in shear.

(1) $$Q_{su,mean}=\left\{\frac{0.115k_{u}k_{p}(18+F_{c})}{M/Qd\;+0.12}+0.85\sqrt{p_{w}\sigma_{wy}}+0.1\sigma_{0}\right\}\;bj$$

In Equation (1), *k_u_* denotes a correction factor related to the effective column depth and was taken as 0.75, while *k_p_* is defined as 0.82*p_t_*^0.23^, where *p_t_* represents the ratio of longitudinal tensile reinforcement (%). The material parameters include the concrete compressive strength *F_c_* (MPa), the transverse reinforcement ratio *p_w_*, the yield strength of transverse reinforcement *σ_wy_* (MPa), and the axial stress *σ*_0_ (MPa). The geometric parameters consist of the column width *b*, which was replaced by the effective width *b*′ in this study to account for the hollow section, the column depth *D*, and the effective depth *d*. The shear span ratio is expressed as *M*/*Q*, and the internal lever arm *j* was calculated as 7/8*d*, corresponding to the distance between the compressive and tensile resultants. For Equation (1), the shear span ratio *M*/*Q* was restricted to the range of 1 to 3. Values smaller than 1 were taken as 1, whereas values exceeding 3 were capped at 3.

In this study, the applicability of hollow columns is examined primarily based on the Japanese shear strength evaluation formulas. For reference, the nominal shear strength (*V_n_*) according to ACI 318 [23] is also evaluated, expressed as $V_{n}\;=\;V_{c}+V_{s}$, with $V_{c}=0.17\lambda \sqrt{{f^{\prime }}_{c}}b_{w}d+\frac{N_{u}}{6A_{g}}b_{w}d$. The results of this supplementary evaluation are presented in Table 3. It should be noted that, in order to reflect the effect of the hollow section, the effective column width (*b*′) was also adopted in place of *b_w_* when calculating *V_n_*. Since the shear strength contributions of concrete and shear reinforcement are defined differently in ACI 318 and AIJ, the calculated shear capacities yielded different values; however, both formulas consistently predicted the same failure mode of the specimens.

### 2.2. Loading Program and Instrumentation

**Figure 3:**
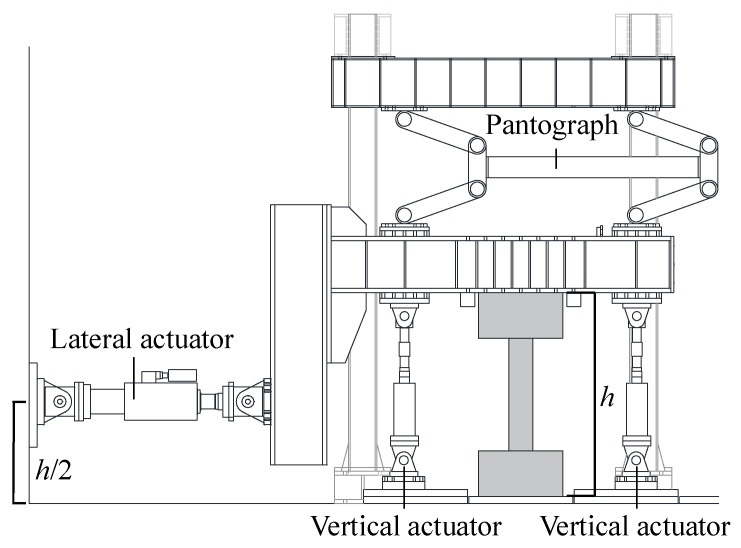
Test setup and loading configuration (example shown for CM3).

The experimental loading configuration is shown in Figure 3. To generate an antisymmetric bending moment along the column, the lateral actuator (Saginomiya Seisakusho, Inc., Tokyo, Japan) was installed at the mid-height of the specimen, with a pantograph system placed above the column. A constant axial load was applied throughout the test using two vertically arranged jacks (Ox Jack Co., Ltd., Tokyo, Japan) positioned symmetrically with respect to the column axis.

Lateral loading was imposed under displacement-controlled conditions, and two loading cycles were applied at each target deformation level in both positive and negative directions. The prescribed lateral drift ratios were 0.25%, 0.5%, 1.0%, 1.5%, 2.0%, 3.0%, and 4.0%. In this study, the lateral drift ratio *R* is defined as the ratio of the lateral displacement to the clear height of the column.

**Figure 4:**
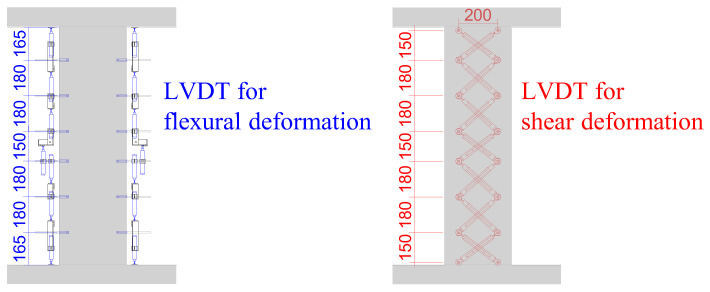
Measurement schemes (example shown for CM3).

Figure 4 illustrates the instrumentation layout adopted in this study. Local flexural and shear deformations along the column height were monitored using displacement transducers (Tokyo Measuring Instruments Laboratory Co., Ltd. (TML), Tokyo, Japan), while the strains in both longitudinal and transverse reinforcement were measured by strain gauges (TML). The experimental observations related to deformation components and reinforcement strain responses are discussed in detail in Section 3.

## 3. Experimental Results and Observations

### 3.1. Load-Deflection Relationship

**Figure 5:**
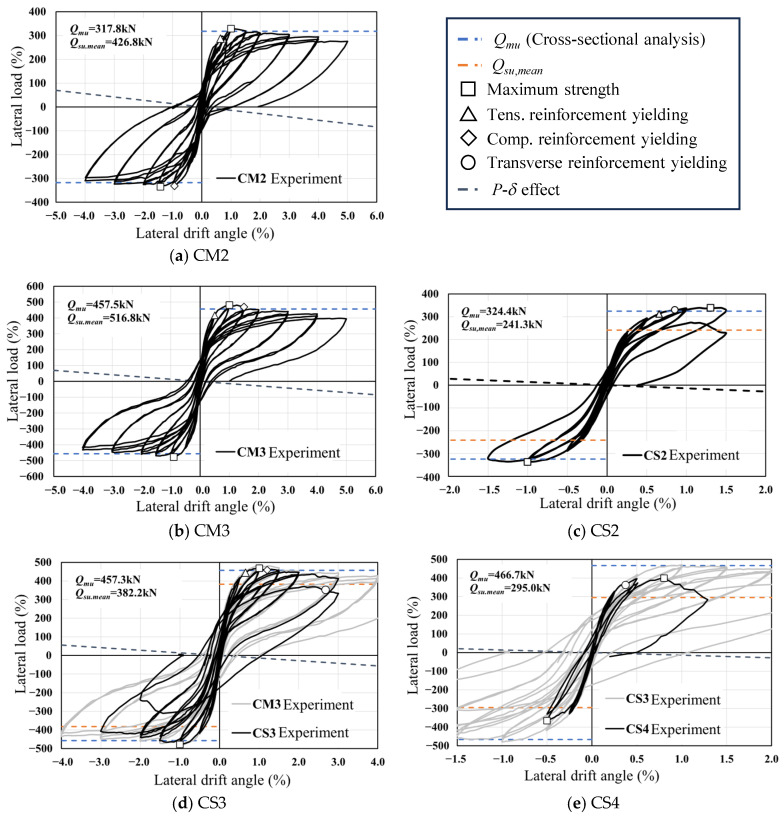
Hysteresis curves obtained from experimental results.

Figure 5 compares the load–deflection responses of all tested specimens together with those of the reference specimen CM2 reported in the previous study [19]. Different markers are used to identify key response points in the hysteresis curves, where □ denotes the peak load, △ and ◇ correspond to yielding of the tensile and compressive longitudinal reinforcement, respectively, and ○ indicates yielding of the transverse reinforcement. The *P*–*δ* line is also plotted in Figure 5. The *P*–*δ* effect was evaluated by calculating the additional moment induced by the applied axial load and lateral displacement (*N*·*δ*), which was then divided by the column height to obtain the corresponding increment in lateral force. Because the axial load remained constant during testing, the resulting *P*–*δ* relationship appears as a straight line. Based on these responses, the main features of the observed hysteretic behavior are summarized below.

For specimen CM3, yielding of the tensile longitudinal reinforcement initiated at a lateral drift ratio of *R* = 0.56%. The peak lateral resistance was attained at drift ratios of *R* = +1.01% and −0.90% under positive and negative loading, respectively. Yielding of the compressive reinforcement was subsequently observed at *R* = +1.46%. Even at a drift ratio of *R* = +5.0%, the lateral load capacity remained greater than 80% of the maximum strength, indicating no pronounced strength degradation. The experimentally measured peak strength exceeded the flexural strength predicted by cross-sectional analysis (*Q_mu_*) by approximately 5%. This close agreement demonstrates that the plane-section-based analytical approach can reliably capture the flexural behavior of RC columns with rectangular hollow cross-sections when flexural failure governs. Comparable hysteretic characteristics and peak strength levels were also identified in the previously tested specimen CM2 and in other specimens exhibiting flexure-dominated responses [19]. Based on the present experimental observations together with the authors’ previous findings [19], the interaction between the applied axial force and the resulting neutral axis position at the ultimate flexural state is recognized as a key parameter governing deformation capacity and structural applicability. When the neutral axis at ultimate flexure is located within the flange region of the hollow section, a flexural failure mode can be expected. Accordingly, it is recommended that the neutral axis position under the design axial force be evaluated at the ultimate flexural state when designing RC columns with rectangular hollow cross-sections.

In specimen CS2, with a shear-to-flexural strength ratio (*Q_su_*_,*mean*_/*Q_mu_*) of 0.74, shear failure was intended to occur before flexural failure. However, tensile yielding of the longitudinal reinforcement was observed at *R* = +0.66%, followed by yielding of the transverse reinforcement at *R* = +0.85%. After these yielding, the lateral strength reached its peak at *R* = −1.0% and +1.3%. During the second loading cycle at *R* = +1.5%, a sudden drop in lateral strength occurred near *R* = +1.0% due to shear failure, leading to the termination of the test. Although the *Q_su_*_,*mean*_/*Q_mu_* ratio of 0.74 indicated that shear failure would precede flexural failure, the load–deformation relationship revealed that shear failure occurred immediately after flexural yielding. The experimentally obtained maximum strength was approximately 1.4 times greater than *Q_su_*_,*mean*_, indicating an overestimation relative to the calculated shear strength. As previously explained, the value of *Q_su_*_,*mean*_ was calculated using the column cross-section width (*b*′) excluding the hollow portion, to consider the effect of the hollow core. Consequently, the flange areas above and below the hollow section were not considered in the calculation, which likely resulted in an underestimation of *Q_su_*_,*mean*_. Nevertheless, it was reconfirmed that Equation (1) provides a conservative estimation of shear strength for RC columns with hollow cross-sections. It should also be noted that in Section 3.5, the shear strength will be re-evaluated based on the equivalent cross-sectional area.

Specimen CS3 was also designed to fail in shear, with a *Q_su_*_,*mean*_/*Q_mu_* ratio of 0.84. However, tensile yielding of the longitudinal reinforcement was observed at *R* = +0.66%. The maximum lateral capacities in both positive and negative directions were recorded at *R* = ±1.0%, followed by compressive yielding of the longitudinal reinforcement at *R* = +1.21% and yielding of the shear reinforcement at *R* = +2.68%. Similar to CS2, flexural failure preceded shear failure in CS3, even though the *Q_su_*_,*mean*_/*Q_mu_* ratio was less than 1.0. This behavior is attributed to the conservative estimation of the effective cross-section width, as previously discussed. As a result, the experimental maximum strength closely matched the ultimate flexural strength *Q_mu_* obtained from the cross-sectional analysis. It is worth noting that, although flexural failure occurred in this specimen, its deformation capacity was smaller than that of CM3, which is considered due to the lower *Q_su_*_,*mean*_/*Q_mu_*.

For specimen CS4, which had the lowest *Q_su_*_,*mean*_/*Q_mu_* ratio of 0.63, a major shear crack appeared at the mid-height of the column, and the shear reinforcement began yielding at *R* = +0.38%. Shear failure occurred at *R* = +0.8% during the loading cycle of *R* = +1.0%, accompanied by a rapid decrease in lateral strength due to the widening of the shear crack. Unlike CS2 and CS3, CS4 exhibited shear failure prior to flexural failure, as expected, owing to its lowest *Q_su_*_,*mean*_/*Q_mu_* ratio. The experimental maximum shear strength was approximately 1.35 times greater than the calculated *Q_su_*_,*mean*_, indicating that while the shear capacity was underestimated, it was still conservatively evaluated. Notably, this ratio was nearly the same as that of CS2 (around 1.4), in which flexural and shear failure occurred almost simultaneously.

Although the primary failure mode of each specimen was classified as either flexural-dominant or shear-dominant based on the shear strength margin, it should be recognized that the seismic behavior of RC columns is generally governed by the interaction between flexural and shear mechanisms rather than by purely distinct failure modes. Previous studies on RC and steel-reinforced concrete composite columns have also reported that coupled shear–flexural interaction plays an important role in determining the overall seismic response of columns subjected to cyclic loading [24,25]. In the present study, several specimens, particularly CS2 and CS3, exhibited flexural yielding prior to the development of significant shear cracking, indicating the presence of mixed shear–flexural behavior rather than completely independent failure mechanisms. From a design perspective, however, the evaluation of both flexural and shear capacities is essential when applying hollow RC columns in order to ensure that flexural failure precedes shear failure. For this reason, the specimens in this study were categorized according to their dominant response characteristics for clarity of discussion. Therefore, the observed structural responses should be interpreted within the broader framework of shear–flexural interaction.

Based on the experimental results, it was confirmed that, under an axial force ratio of 0.15, the flexural strength of RC members with hollow cross-sections can be accurately predicted using conventional flexural theory based on the plane-section assumption. Additionally, it was found that Equation (1) proposed by Arakawa [20] provides a conservative estimate of their shear capacity. Consistent with this observation, the nominal shear strength (*V_n_*) obtained from ACI 318 [23] also exhibited a similar tendency.

### 3.2. Failure Pattern

**Figure 6:**
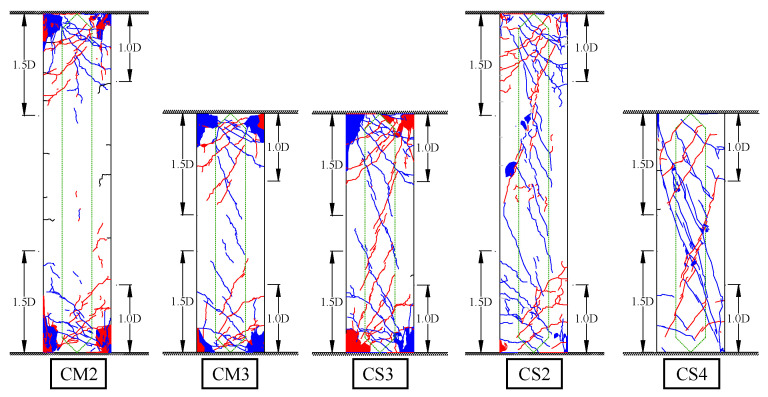
Failure patterns at final loading stages.

Figure 6 illustrates the crack patterns of each specimen at the final loading stage. In the figure, blue and red lines indicate cracks formed during positive and negative loading, respectively, while green lines denote the hollow core regions of the column.

Compared to CM2, shear cracks were observed near the center of the column height in CM3, which were not present in CM2. In addition, the damage caused by flexural deformation near the critical section in CM3 was concentrated in a narrower region than in CM2. This is attributed to the relatively increased contribution of shear deformation due to the reduced column height. Although some shear cracks developed in CM3, their widths were limited to approximately 0.03 to 0.04 mm at peak loading and reduced to around 0.01 mm upon unloading, suggesting that these cracks had little impact on the structural performance. Compared to CM3, CS3 also exhibited flexural failure as the primary failure mode, resulting in a similar damage pattern, such as crushing and spalling of the cover concrete near the critical section. However, while shear cracks near the center of the column height were observed at *R* = 1.0% in CM3, they appeared earlier at *R* = 0.5% in CS3. Moreover, the development and severity of shear crack damage in CS3 were more pronounced than those in CM3.

Compared to CS2, CS4 exhibited more prominent and earlier shear crack formation due to the short column effect. In CS4, shear cracks were dominant, while flexural cracks were less significant than those observed in CS2. Furthermore, the shear crack widths in CS4 were substantially larger than those in CS2. It should be noted that CS2 served only as a reference specimen, as flexural failure preceded shear failure. From the above observations, it was confirmed that reducing the shear span ratio by shortening the column height leads to an increase in shear behavior, as indicated by the development of shear cracks and the concentration of flexural crack formation within a narrower region near the critical section.

### 3.3. Flexural and Shear Deformation Behavior

**Figure 7:**
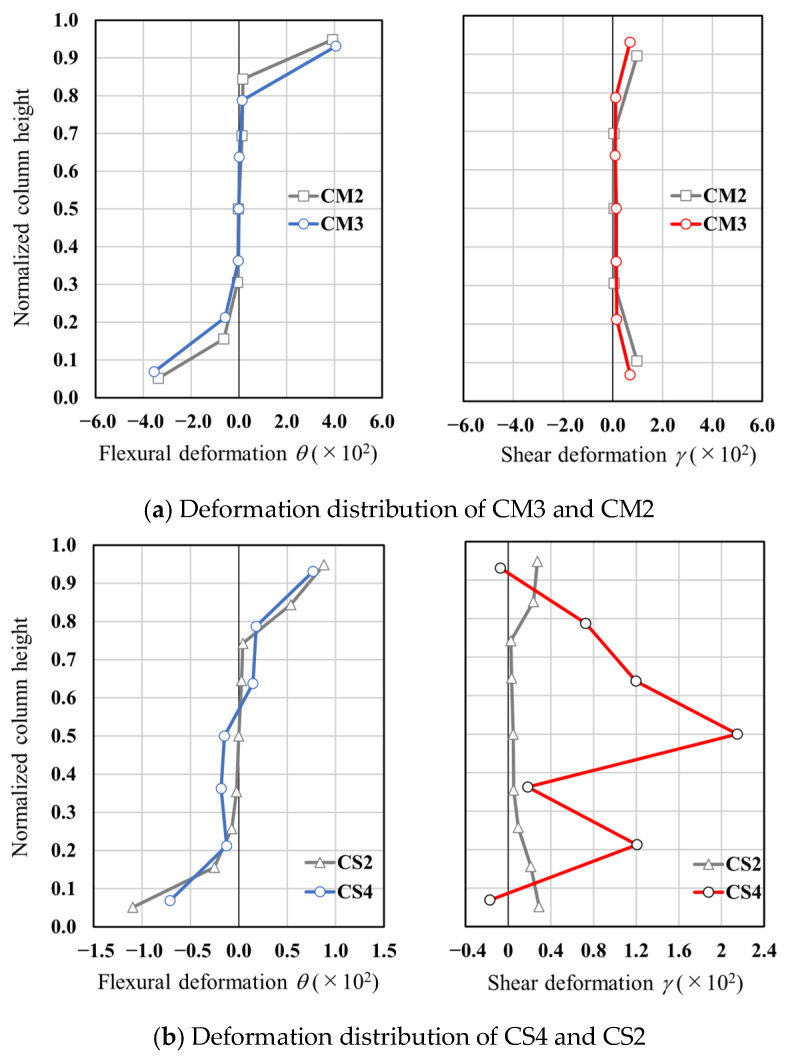
Comparison of flexural and shear deformation distributions.

Figure 7 presents the distributions of flexural and shear deformations for specimens CM3 and CM2 at a drift ratio of *R* = +4.0%, as well as for specimens CS4 and CS2 at *R* = +1.29%, which corresponds to the onset of shear failure in CS4. The evaluation procedure for flexural and shear deformations is described below.

**Figure 8:**
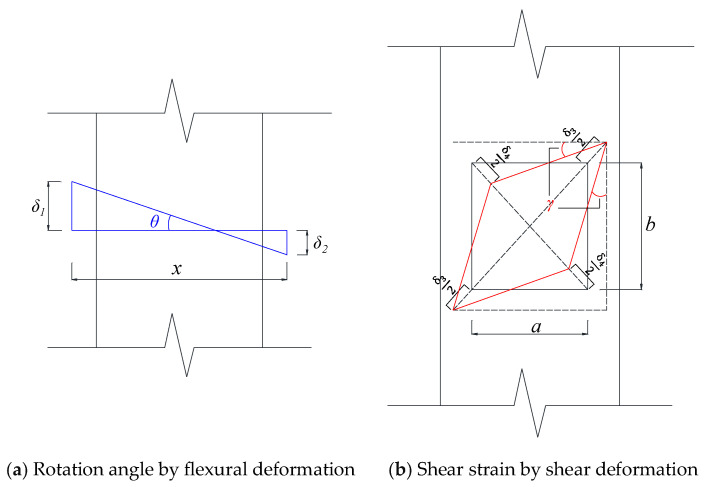
Method for calculating flexural rotation and shear strain.

As described earlier, the column height was discretized into multiple segments. For each segment, the flexural rotation angle (*θ*) and the shear strain (*γ*) were evaluated. Based on the geometrical relationships illustrated in Figure 8, the values of *θ* and *γ* were calculated using Equations (2) and (3), respectively.

With respect to flexural deformation, the distributions along the column height were generally similar between CM3 and CM2, as well as between CS4 and CS2. In contrast, a pronounced difference was observed in shear deformation at the mid-height of the column. Specifically, the shear deformation in CM3 was approximately 2.6 times larger than that in CM2, while CS4 exhibited a shear deformation about 45.4 times greater than that of CS2.

(2) $$\theta =\frac{\delta_{1}-\delta_{2}}{x}$$

(3) $$\gamma =\frac{\sqrt{a^{2}+b^{2}}}{2ab}(\delta_{3}+\delta_{4})$$

In these expressions, *x* represents the spacing between adjacent LVDT measurement points. The symbols *δ*_1_ and *δ*_2_ denote the vertical displacement increments measured by the corresponding LVDTs. The parameters a and b indicate the horizontal and vertical distances between the measurement points, respectively, while *δ*_3_ and *δ*_4_ correspond to the displacement increments recorded by the LVDTs installed in the diagonal direction.

### 3.4. Strain Distribution of Reinforcement

**Figure 9:**
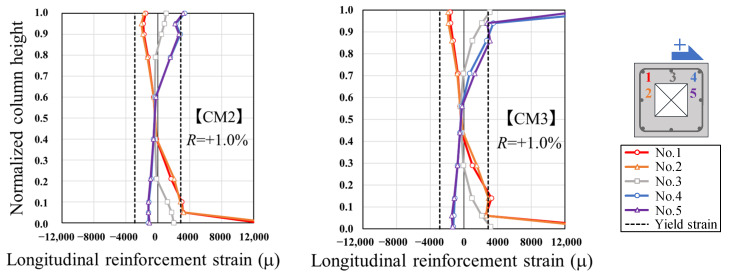
Strain distributions in longitudinal reinforcement (*R* = +1.0%).

Figure 9 presents the strain distribution in the longitudinal reinforcement of CM3 and CM2 at a drift angle of *R* = +1.0%, corresponding to the stage just after the peak lateral strength. As shown in the figure, strain gauges were attached to five positions of the longitudinal reinforcement to examine the effect of the hollow cross-section on strain variation. The strain distributions at positions 1, 2, 3, 4, and 5 are represented by red, orange, gray, blue, and purple lines, respectively. The vertical axis was normalized to 1.0 to account for the difference in column height between CM3 and CM2. The strain values at gauge positions 1 and 2, and at 4 and 5 in CM3 were nearly identical, indicating almost no influence from the presence of the hollow section. Furthermore, the overall strain distributions along the column height in both CM3 and CM2 were very similar, except for localized yielding strains near the critical section. These results suggest that the variation in shear span ratio had little effect on the longitudinal strain behavior.

**Figure 10:**
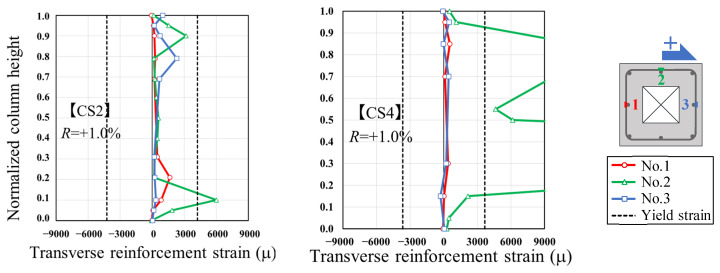
Strain distributions in transverse reinforcement for CS2 and CS4.

Figure 10 illustrates the strain distribution of the transverse reinforcement in CS2 and CS4 at *R* = +1.0%. It should be noted that CS2 primarily exhibited flexural failure, while CS4 failed in shear. Strain gauges were attached to positions 1, 2, and 3 of the transverse reinforcement, as shown in the figure, to investigate the influence of the hollow cross-section. The vertical axis in Figure 10 is normalized to 1.0, as the column heights of the two specimens differ. Comparing CS4 and CS2, after *R* = 0.5%, CS2 exhibited increasing strain values at the top and bottom of the column, whereas CS4 showed increased strain values at the mid-height of the column. This behavior is consistent with the previously described shear deformation distribution and aligns with the cross-sectional locations where shear failure was observed.

### 3.5. Reevaluation of Shear Capacity for RC Column with Hollow Cross-Sections

**Figure 11:**
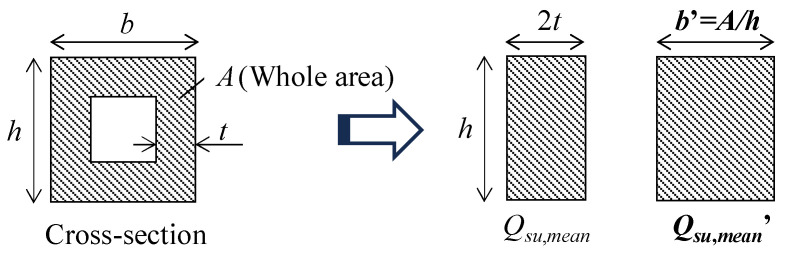
Substitution of hollow section with equivalent section for calculating *Q_su_*_,*mean*_′.

When calculating the shear strength *Q_su_*_,*mean*_ of RC columns using Equation (1) proposed by Arakawa [20], the entire beam width (*b*) is used. Similarly, in accordance with ACI 318 [23], the nominal shear strength (*V_n_*) is also calculated using the entire beam width (*b_w_*). However, to account for the influence of the hollow portion, in this study, the flange areas located above and below the hollow section were excluded during the design stage, which likely led to an underestimation of the shear capacity. To address this, the full cross-section excluding the hollow portion, was replaced with an equivalent solid section having the same column depth. This approach enables consideration of the shear resistance provided by the flange regions. The concept of this equivalent replacement is illustrated in Figure 11. In other words, the equivalent column width *b*′ was calculated by dividing the total cross-sectional area by the column depth, and this value was used in Equation (1) to recalculate the shear strength.

The recalculation results for all specimens using Equation (1) are summarized in Table 4. As shown in the table, the experimental results correspond well with the recalculated values of *Q_su_*_,*mean*_/*Q_mu_*. It should be noted that when *Q_su_*_,*mean*_/*Q_mu_* exceeds 1.0, the column exhibits flexural failure, whereas shear failure occurs when the value is below 1.0. Although the number of hollow-sectioned specimens that primarily failed in shear is limited, these results indicate that the failure modes of all specimens were accurately predicted using the equivalent column width *b*′, derived from the total cross-sectional area.

The failure mode of each specimen was identified based on the comparison between calculated flexural strength (*Q_mu_*) and shear strength (*Q_su_*_,*mean*_), together with observed cracking patterns and reinforcement yielding sequence. Specimens were classified as flexural-dominant when flexural yielding preceded significant shear degradation, whereas shear-dominant behavior was defined when shear cracking and strength deterioration governed the response before full flexural capacity development. The results further indicate that the equivalent cross-sectional area approach improves the prediction accuracy of shear capacity for hollow RC columns and provides a rational basis for practical design application.

In addition, the shear margin was evaluated using the nominal shear strength *V_n_* obtained from ACI 318 as a reference value, expressed as *V_n_*/*Q_mu_*. The outcomes of this supplementary evaluation are summarized in Table 5. It should be noted that *b*′ was also employed in the ACI 318 equation for the recalculation of shear strength. The results were generally comparable to those in Table 4 based on the AIJ formulas; however, for certain specimens, such as CS2, discrepancies were observed where the calculated shear margin did not coincide with the experimental results. These observations suggest that while the ACI 318 provisions can provide a reasonable reference for estimating shear margins, caution is required when applying them to hollow RC columns. Further experimental and analytical studies are needed to validate the shear strength prediction method for RC members with hollow cross-sections.

It is noted that some international standards, such as Eurocode 8 [26], consider the beneficial contribution of axial load due to self-weight in the evaluation of shear resistance. In such approaches, axial compression may enhance shear capacity under certain conditions. Although the present study primarily evaluates shear strength based on Japanese and American formulations, the relatively low axial force ratio adopted in this study suggests that similar trends would be expected under Eurocode provisions. A detailed comparison is beyond the scope of the present work but remains an interesting topic for future investigation.

## 4. Finite Element (FEM) Modeling and Analysis

### 4.1. Finite Element Model Overview

**Figure 12:**
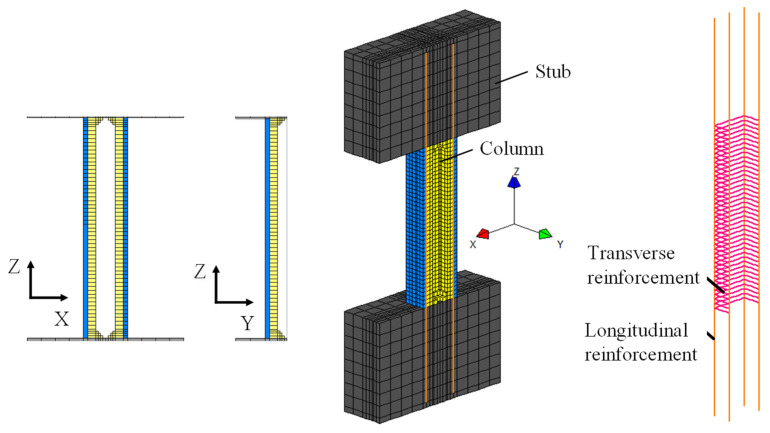
FEM model geometry and meshing configuration (example shown for CM3).

To gain further insight into the seismic response of the test specimens, a nonlinear finite element analysis was performed. The analysis was conducted using the program FINAL (ver.11) [27], which has been extensively applied to reinforced concrete structures in Japan. The configuration of the numerical model, including the geometry and mesh discretization, is shown in Figure 12.

In the model, concrete was discretized using three-dimensional hexahedral elements, whereas the longitudinal and transverse reinforcements were modeled with truss elements. The interaction between concrete and reinforcement was represented by incorporating bond–slip behavior. The reinforcing bars were explicitly modeled as discrete truss elements embedded within the concrete solid elements. The interaction between reinforcement and surrounding concrete was represented through bond–slip constitutive relationships assigned along the reinforcement elements, rather than assuming perfect bond. Owing to the use of hexahedral elements, the tapered hollow region was modeled through a finely stepped approximation, as illustrated in Figure 12.

To improve computational efficiency, symmetry was exploited by modeling only half of the specimen, with translational displacement in the Y direction constrained along the symmetry plane. The axial load was applied as distributed forces in the Y direction at the top surface of the upper stub, and lateral displacement was imposed at the same location to reproduce the experimental loading conditions.

### 4.2. Constitutive Model of the Material

**Figure 13:**
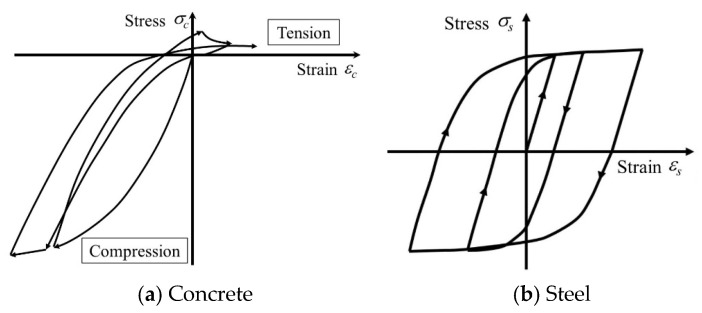
Hysteretic models for concrete and reinforcing steel.

**Figure 14:**
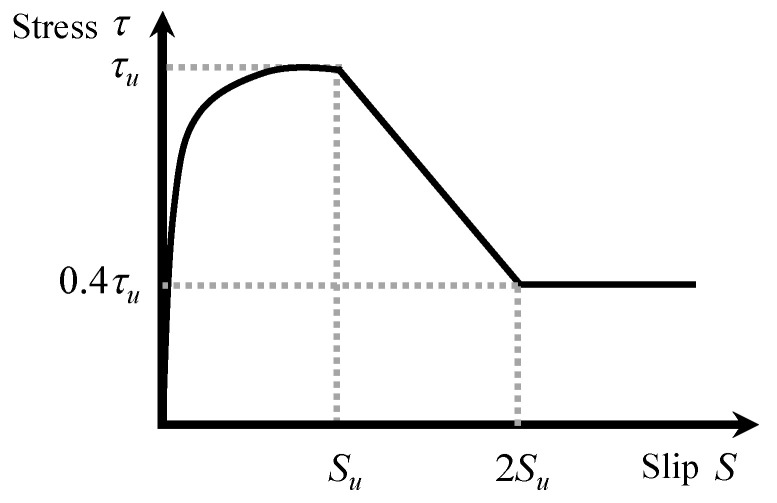
Bond-slip model between reinforcement and concrete.

The constitutive relationships adopted for concrete, reinforcing steel, and the bond–slip interaction are summarized in Figure 13 and Figure 14. For concrete, the cyclic stress–strain response was modeled using the hysteresis formulation proposed by Naganuma et al. [28]. In the ascending branch up to the peak stress, the modified Ahmad model [29] was employed. The post-peak softening behavior was represented using different constitutive descriptions for the core and cover concrete: the Nakamura–Higai model [30] was applied to the confined core region, whereas a model characterized by rapid stress degradation beyond the peak was assigned to the cover concrete [27].

Concrete failure under triaxial stress states was evaluated using Ottosen’s four-parameter failure criterion [31], with material coefficients calibrated according to the recommendations by Hatanaka [32]. In tension, concrete was assumed to behave elastically up to crack initiation, after which tensile softening was described following the model proposed by Izumo et al. [33].

The cyclic behavior of reinforcing steel was simulated using the modified Menegotto–Pinto model [34]. The corresponding envelope curve was defined by a bilinear relationship, and the post-yield stiffness was set to 1/1000 of the elastic modulus *E_s_*. The bond–slip relationship between the longitudinal reinforcement and surrounding concrete was modeled in accordance with the formulations proposed by Naganuma et al. [35] and the AIJ Guidelines [36].

### 4.3. Numerical Analysis Results

**Figure 15:**
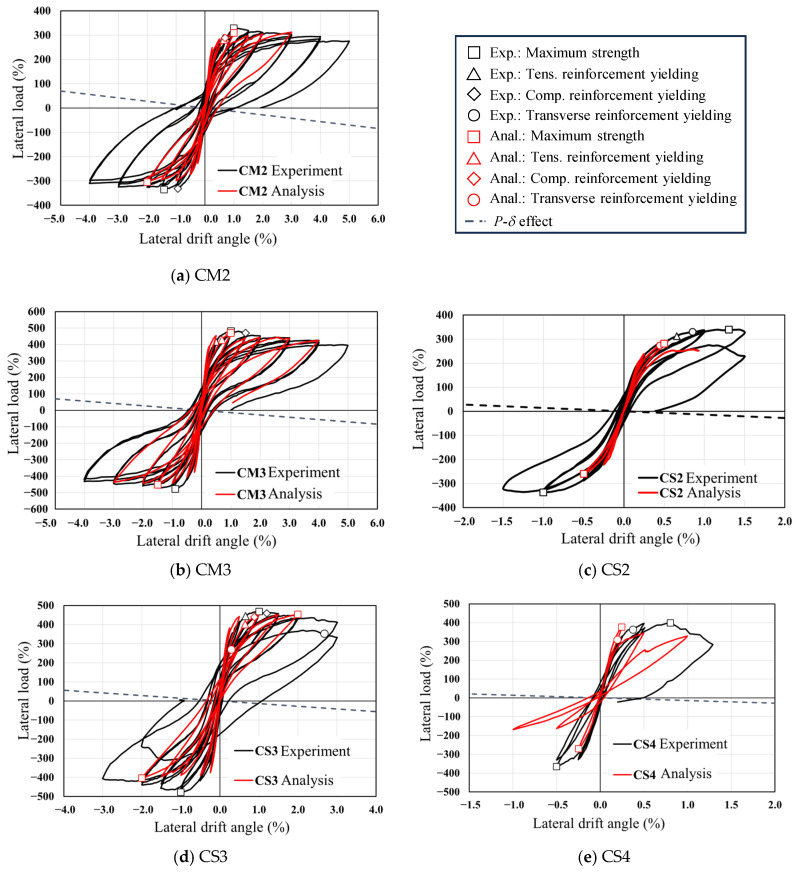
Comparison of hysteresis curves from experiments and FEM analyses.

Figure 15 presents a comparison between the hysteresis responses obtained from the experimental tests and those predicted by the FEM analysis. The numerical results are shown up to the drift level at which the peak strength was approximately attained, within a range where stable numerical convergence was maintained. Distinct symbols are used in the figure to indicate key response points: □ corresponds to the maximum strength, △ and ◇ represent yielding of the tensile and compressive longitudinal reinforcement, respectively, and ○ denotes yielding of the transverse reinforcement.

For CM2 and CM3, in which flexural failure was the dominant mechanism, the initial stiffness and the tensile yielding behavior of the main reinforcement closely matched the experimental results. The maximum load-bearing capacities also showed good agreement, with the analytical values being approximately 0.95 to 0.98 times those observed in the experiments, indicating a conservative estimation. Although minor discrepancies were observed in the peak strength and the compression reinforcement yielding points, the overall restoring force behavior was well captured, demonstrating that the FEM analysis reliably reproduced the load-deformation responses of CM2 and CM3. As shown in Figure 15, in the cases of the specimens, where shear failure was expected to be the primary failure mode, a noticeable reduction in load-bearing capacity was observed in the analysis results, particularly under negative loading. A detailed review of the analysis revealed that, following shear cracking in the central region of the column during positive loading, the compressive stress could no longer be sustained in the hollow cross-section regions during the subsequent negative loading cycle. Consequently, the lateral strength did not increase significantly under negative loading. Enhancing the accuracy of the FEM analysis for shear-failure-dominant specimens remains a subject for future work. Nevertheless, under positive loading, the load-deformation envelopes were generally well reproduced, with conservative predictions by the analysis.

## 5. Conclusions

In this study, experimental and analytical investigations were carried out to gain a better understanding of the structural behavior of RC columns with hollow cross-sections. The key findings of this study are summarized as follows.

(1) In specimen CM3, which failed primarily in flexure, shear-related behavior and responses were observed to increase due to the reduction in shear span ratio from 2.79 (in CM2) to 1.97. Nevertheless, similar to CM2, no significant degradation in load-bearing capacity was observed even at large deformations, and stable load-deformation behavior was confirmed.
(2) For specimens that experienced flexural failure, the maximum strength by experiments was approximately 1.05 times greater than the predicted strength by cross-sectional analysis. This confirms that conventional flexural theory accurately predicts the flexural strength of RC columns with hollow cross-sections. In addition, longitudinal reinforcement strains measured at the same height showed negligible influence from the presence of the hollow section.
(3) Regarding the shear strength formula proposed by Arakawa, the experimental maximum shear capacities were approximately 1.35 to 1.40 times higher than the calculated values. This indicates that the shear strength of RC columns with hollow cross-sections is conservatively estimated, although somewhat underestimated.
(4) To improve the accuracy of shear strength evaluation, the entire hollowed cross-section was replaced with an equivalent solid cross-section having the same depth. This equivalent section was then applied in Arakawa’s shear strength formula. As a result, the experimentally observed failure modes aligned well with the recalculated shear strength margins (*Q_su_*_,*mean*_/*Q_mu_*), although further validation is required with additional specimens. In addition, the nominal shear strength according to ACI 318 was also examined as a reference value, and the corresponding shear margins (*V_n_*/*Q_mu_*) were found to be generally consistent with those obtained using the AIJ formulas, despite some discrepancies in certain specimens.
(5) In terms of reproducing the experimental results, the nonlinear FEM analysis successfully captured the maximum load-bearing capacity, hysteresis behavior, and yielding of reinforcement for specimens governed by flexural failure. However, for specimens dominated by shear failure, the analysis did not fully capture the load-deformation behavior under negative loading. Nevertheless, the load-deformation envelope under positive loading was reasonably well reproduced, providing conservative estimates.

As previously discussed, it should be emphasized that the location of the neutral axis at the ultimate flexural state is an important factor in ensuring flexural failure modes and achieving ductile behavior. In the test specimens of this study, which had an axial force ratio of 0.15, the neutral axis at the ultimate flexural state was located within the flange of the hollow cross-section. Therefore, checking the position of the neutral axis under the design axial force is recommended when adopting hollow RC columns.

In typical seismic design scenarios where flexural hinges are expected to form at beam ends, rectangular hollow RC columns may be more suitably applied to upper-story columns, where column hinge formation is less likely. These findings highlight the potential applicability of hollow RC columns in seismic design, provided that both flexural and shear behaviors are carefully considered, although further refinement of numerical models is required to improve the prediction accuracy of shear-dominated responses.

From a design perspective, the relative margin between flexural and shear capacities can be used to anticipate the dominant failure mode of hollow RC columns. It is recommended that the shear capacity of hollow columns be evaluated using the equivalent cross-sectional area approach, which enhances the reliability and conservatism of shear strength prediction in practical design.

Future research should further investigate the influence of material degradation and long-term deterioration on the seismic performance of hollow RC columns. In particular, the interaction between the conservation state of the section and shear–flexural behavior warrants additional study. Comparative analyses under different degradation scenarios would provide valuable insight into the long-term reliability of hollow-sectioned columns in seismic regions.

## Figures and Tables

**Table 1:** Summary of specimen properties.

Specimen	Previous Study	This Study			
	CM2	CS2	CM3	CS3	CS4
**Cross-Section**	340 mm × 340 mm				
**Hollow Cross-Section**	150 mm × 150 mm				
**Hollow Section Ratio**	0.81				
**Height**	1600 mm		1200 mm		
**Longitudinal Reinforcement**	8-D13(SD490)				
**Transverse Reinforcement**	U9.0@30 (SBPD1275)	D4.0@60 (SD295A)	U10.7@30 (SBPD1275)	D10@60 (UHY685)	D4@60 (SD295A)
Stirrup volumetric ratio *^a^	0.009%	0.001%	0.013%	0.005%	0.001%
**Axial Force**	1400 kN				
**Axial Force Ratio**	0.164	0.148	0.144	0.146	0.143
**Concrete Compressive Strength**	91.8 MPa	101.8 MPa	104.1 MPa	102.8 MPa	105.5 MPa
**Ultimate Flexural Strength *Q_mu_***	317.8 kN	324.4 kN	457.5 kN	457.3 kN	466.7 kN
**Shear Strength *Q_su_*_,*mean*_**	426.8 kN	241.3 kN	516.8 kN	382.2 kN	295.0 kN
***Q_su_*_,*mean*_** **/*Q_mu_***	1.34	0.74	1.13	0.84	0.63
**Expected Primary Failure Mode**	Flexural	Shear	Flexural	Shear	Shear

*^a^ Stirrup volumetric ratio (*ρ_v_*) was calculated as *ρ_v_* = *A_sh_*/(*s*·*A_c_*), where *A_sh_* is the total cross-sectional area of stirrups within spacing *s* (four legs), and *A_c_* is the net concrete area excluding the hollow core.

**Table 2:** Mechanical properties of reinforcement materials.

	Yield Strain (μ)	Yield Strength (MPa)
**D13 (SD490)**	2944	537.0
**D10 (UHY685)**	3907	826.8
D4 (SD295A) *^a^	3663	339.8
U10.7 (SBPD1275) *^a^	8556	1294.1
U9.0 (SBPD1275) *^a^	8884	1373.0

*^a^ Strength and strain by 0.2% offset method.

**Table 3:** Nominal shear strength based on ACI 318 (Reference values).

Specimen	Previous Study	This Study			
	CM2	CS2	CM3	CS3	CS4
**Ultimate Flexural Strength *Q_mu_***	317.8	324.4	457.5	457.3	466.7
**Nominal Shear Strength *V_n_***	685.0	187.8	906.7	443.4	187.8
***V_n_*** **/*Q_mu_***	2.16	0.58	1.98	0.97	0.40
**Expected Primary Failure Mode**	Flexural	Shear	Flexural	Shear	Shear

**Table 4:** Recalculated shear strength margin (*Q_su_*_,*mean*_/*Q_mu_*) and primary failure modes.

Specimen	Previous Study	This Study			
	CM2	CS2	CM3	CS3	CS4
**Ultimate Flexural Strength *Q_mu_***	317.8	324.4	457.5	457.3	466.7
**Recalculated Shear Strength *Q_su_*_,*mean*_′**	614.6	347.5	744.2	550.4	424.8
***Q_su_*_,*mean*_** **′/*Q_mu_***	1.93	1.07	1.63	1.21	0.91
**Primary Failure Mode by Recalculation**	**Flexural**	**Flexural**	**Flexural**	**Flexural**	**Shear**
**Primary Failure Mode by Experiment**	**Flexural**	**Flexural**	**Flexural**	**Flexural**	**Shear**

**Table 5:** Recalculated shear strength margin (*V_n_*/*Q_mu_*) and primary failure modes (Reference (ACI 318) values).

Specimen	Previous Study	This Study			
	CM2	CS2	CM3	CS3	CS4
**Ultimate Flexural Strength *Q_mu_***	317.8	324.4	457.5	457.3	466.7
**Recalculated Nominal Shear Strength *V_n_*** **′**	746.5	249.3	968.2	504.9	249.3
***V_n_*** **′/*Q_mu_***	2.35	0.77	2.11	1.10	0.53
**Primary Failure Mode by Recalculation**	**Flexural**	**Shear**	**Flexural**	**Flexural**	**Shear**
**Primary Failure Mode by Experiment**	**Flexural**	**Flexural**	**Flexural**	**Flexural**	**Shear**

## Data Availability

The original contributions presented in this study are included in the article. Further inquiries can be directed to the author.
